# Intra-amniotic Infection Caused by *Capnocytophaga* Species

**DOI:** 10.1155/S1064744996000580

**Published:** 1996

**Authors:** Hiroshige Mikamo, Kyoko Kawazoe, Yasumasa Sato, Koji Izumi, Teruhiko Tamaya

**Affiliations:** Department of Obstetrics and Gynecology School of Medicine Gifu University Gifu City Gifu 500 Japan

## Abstract

*Background: Capnocytophaga* species are common oral pathogens and infrequent causes of systemic infection in patients with compromised host. The isolation of this organism suggests an oral source of infection.

*Case:* A 32-year-old woman was admitted at 23 weeks gestation in preterm premature rupture of the membranes. She subsequently developed signs of clinical intra-amniotic infection, including fever, fetal tachycardia, and uterine tenderness. Bacteriologic studies of the amniotic fluid by trans-abdominal amniocentesis and subchorionic placental cultures yielded *Capnocytophaga* species. On review of the patient's history, a temporal relation was noted between orogenital contact and onset of clinical infection. Thorough evaluation of the patient, including dental examination with periodontal cultures, did not reveal an obvious source of infection. However, significant periodontal disease was identified in her partner and *Capnocytophaga* species were isolated from her partner.

*Conclusion:* This case suggests that intra-amniotic infection may have been due to an ascending infection after orogenital contact.


Infectious Diseases in Obstetrics and Gynecology 4:301–302 (1996)
© 1997 Wiley-Liss, Inc.

### Intra-Amniotic Infection Caused by *Capnocytophaga* Species

**Hiroshige Mikamo,* Kyoko Kawazoe, Yasumasa Sato, Koji Izumi, and Teruhiko Tamaya**

*Department of Obstetrics and Gynecology, School of Medicine, Gifu University, Gifu, Japan*

### Abstract

***Background:*** **Capnocytophaga species are common oral pathogens and infrequent causes of systemic infection in patients with compromised host. The isolation of this organism suggests an oral source of infection.**

***Case:*** **A 32-year-old woman was admitted at 23 weeks gestation in preterm premature rupture of the membranes. She subsequently developed signs of clinical intra-amniotic infection, including fever, fetal tachycardia, and uterine tenderness. Bacteriologic studies of the amniotic fluid by transabdominal amniocentesis and subchorionic placental cultures yielded *Capnocytophaga* species. On review of the patient's history, a temporal relation was noted between orogenital contact and onset of clinical infection. Thorough evaluation of the patient, including dental examination with periodontal cultures, did not reveal an obvious source of infection. However, significant periodontal disease was identified in her partner and *Capnocytophaga* species were isolated from her partner.**

***Conclusion:*** **This case suggests that intra-amniotic infection may have been due to an ascending infection after orogenital contact. Infect. Dis. Obstet. Gynecol. 4:301–302, 1996.**

© 1997 Wiley-Liss, Inc.

Key words

*Capnocytophaga* species; amniotic infection; orogenital contact

Preterm premature rupture of the membranes (PROM) comprises 1–2% of all pregnancies and is responsible for at least one third of all preterm births.[1] In a recent study, the common bacterial pathogens isolated from chorioamnionitis appeared to be *Streptococcus agalactiae, Escherichia coli, Prevotella* species, and *Fusobacterium* species.[2]

A group of gliding bacteria classified in the genus *Capnocytophaga* species has attracted considerable attention.[3] These organisms are of facultatively anaerobic, capnophilic, fusiform, gram-negative rods. Gliding motility on the surface of some agars is unique as their characteristic.[3]

*Capnocytophaga* appears to be causative of periodontal lesions in idiopathic juvenile periodontitis, whereas it is commonly present in the normal gingival sulcus, independent of the presence of periodontal diseases.[4]

We report a case of intra-amniotic infection caused by *Capnocytophaga* species.

### CASE REPORT

A 32-year-old Japanese woman, gravida 2, para 2, suffered from preterm PROM at 23 weeks gestation. She had no notable past history except cystectomy for a left ovarian cyst at 29 years of age. Laboratory examinations showed leukocyte count 9,900/μl and C-reactive protein 2.5 mg/dl. Tocolysis with ritodrine hydrochloride (50 μg/min ~200 μg/min) and antibacterial chemotherapy of 2 g of flomoxef sodium (FMOX), sodium(-)-(6*R*, 7*R*)-7-[2-(difluoromethylthio)acetamido]-7-methoxy-3-[[1-(2-hydroxyethyl)-1*H*-tetrazol-5-yl]thiomethyl]-8-oxo-5-oxa-1-azabicyclo [4.2.0] oct-2-ene-2-carboxylate, b.i.d., were started for her. However,

*Correspondence to: Hiroshige Mikamo, Department of Obstetrics and Gynecology, School of Medicine, Gifu University, 40, Tsukasa-machi, Gifu City, Gifu 500, Japan.

Received 19 June 1996

Accepted 14 January 1997

her uterine contraction continued. Subsequently, she experienced a mild spike fever of 37.3°C, uterine tenderness, and fetal tachycardia. Amniocentesis yielded slightly cloudy fluid with gram-negative, filamentous rods and leukocytes. She showed rigors and a mild spike temperature of 37.6°C. Treatment of FMOX and ritodrine hydrochloride still continued. However, labor failed to stop and the amniotic fluid became more purulent and foul-smelling. The fetus began to show acute fetal distress, therefore the patient was delivered of a 1,298 g live-born female infant with an Apgar score of 5 by cesarean section at 26 weeks 2 days gestation. The baby developed respiratory distress syndrome, which was controlled afterward. The patient became uneventful rapidly after delivery and was discharged on the 11th postoperative day.

Both placental and amniotic fluid cultures grew *Capnocytophaga* species, as identified by Rap ID ANA System II (Innovative Diagnostic System, Inc., Atlanta, GA). In follow-up, the patient revealed no infectious signs, but stated a history of repeated orogenital contact during her pregnancy, in 1–2 weeks before presentation of preterm PROM. Thorough evaluation of the patient, including dental examination with periodontal cultures, did not reveal an obvious source of infection. Dental examination of her partner with periodontal cultures showed symptomatic periodontal disease, which was caused by *Capnocytophaga* species.

### DISCUSSION

Preterm delivery is the leading cause of perinatal fetal death. Preterm deliveries are associated mostly with preterm PROM.[1] An ascending infection is common for genital tract infections, such as infection to the amniotic cavity. Both aerobes and anaerobes are involved in cases of intra-amniotic infection.[2]

*Capnocytophaga* species are micro-aerophilic gram-negative bacilli. The mouth is a major habitat of these organisms, which are causative for periodontal infections. Clinical infections by these organisms generally are limited to the oral cavity or upper respiratory tract.[5] Bacteria with *Capnocytophaga* species rarely have been reported in immunocompromised or neutropenic patients.[6] Although rarely isolated from the genital tract, there are several case reports about infections caused by *Capnocytophaga* species, with no history of prior orogenital contact.[7,8]

Ampicillin, penicillin, or clindamycin would be the best antibiotics of choice in the therapy of documented *Capnocytophaga* species infection.

In this case, isolation of *Capnocytophaga* species from her partner with periodontal disease and history of orogenital contact brought about her evidence.

This case demonstrates that intra-amniotic infection might occur due to orogenital contact and subsequent ascending infection from the genital tract. Further investigation is needed to grade the pathogenicity of *Capnocytophaga* species.

### REFERENCES

1. Gibbs RS, Blanco JD: Premature rupture of membranes. Obstet Gynecol 60:671–675, 1982.
2. Mikamo H, Ito K, Tamaya T: Studies on the clinical implication of anaerobes, especially *Prevotella bivia* in obstetrics and gynecology. Acta Sch Med Univ Gifu 42: 230–248, 1994.
3. Socransky SS, Holt SC, Leadbetter ER, Tanner ACR, Savitt E, Hammond BF: *Capnocytophaga:* A new genus gram-negative gliding bacteria. III. Physiological characterization. Arch Microbiol 122:29–33, 1979.
4. Newman MG, Socransky SS, Savitt ED, Propas DA, Crawford A: Studies of the microbiology of periodontosis. J Periodontol 47:373–379, 1976.
5. Parenti DM, Snydman DR: *Capnocytophaga* species: Infections in nonimmunocompromised and immunocompromised hosts. J Infect Dis 151:140–147, 1985.
6. Verghese A, Franzus B, Berk S, Roscoe DL, Clarke AM: Antimicrobial susceptibility of *Capnocytophaga* spp. Antimicrob Agents Chemother 37:1206, 1993.
7. Ernest JM, Wasilauskas B: *Capnocytophaga* in the amniotic fluid of a woman in preterm labor with intact membranes. Am J Obstet Gynecol 153:648–649, 1985.
8. Iralu JV, Roberts D, Kazanjian PH: Chorioamnionitis caused by *Capnocytophaga:* Case report and review. Clin Infect Dis 17:457–461, 1993.
